# Gallic acid caused cultured mice TM4 Sertoli cells apoptosis and necrosis

**DOI:** 10.5713/ajas.18.0317

**Published:** 2018-10-26

**Authors:** Wanhong Li, Xiangpeng Yue, Fadi Li

**Affiliations:** 1 State Key Laboratory of Grassland Agro-ecosystems, College of Pastoral Agriculture Science and Technology, Lanzhou University, Lanzhou 730020, China; 2 Key Laboratory of Grassland Livestock Industry Innovation, Ministry of Agriculture and Rural Affairs, Lanzhou University, Lanzhou 730020, China; 3 Engineering Research Center of Grassland Industry, Ministry of Education; Lanzhou University, Lanzhou, 730020, China; 4 Biotechnology Engineering Laboratory of Gansu Meat Sheep Breeding, Minqin, 733300, China

**Keywords:** Apoptosis, Gallic Acid, TM4 Sertoli Cell

## Abstract

**Objective:**

The study was designed to determine the cytotoxic effect of gallic acid (GA), obtained by the hydrolysis of tannins, on mice TM4 Sertoli cells apoptosis.

**Methods:**

In the present study, non-tumorigenic mice TM4 Sertoli cells were treated with different concentrations of GA for 24 h. After treatment, cell viability was evaluated using WST-1, mitochondrial dysfunction, cells apoptosis and necrosis was detected using JC-1, Hoechst 33342 and propidium iodide staining. The expression levels of Cyclin B1, proliferating cell nuclear antigen (PCNA), Bcl-2-associated X protein (BAX), and Caspase-3 were also detected by quantitative real-time polymerase chain reaction and Western-blotting.

**Results:**

The results showed that 20 to 400 μM GA inhibited viability of TM4 Sertoli cells in a dose-dependent manner. Treatment with 400 μM GA significantly inhibited PCNA and Cyclin B1 expression, however up-regulated BAX and Caspase-3 expression, caused mitochondrial membrane depolarization, activated Caspase-3, and induced DNA damage, thus, markedly increased the numbers of dead cells.

**Conclusion:**

Our findings showed that GA could disrupt mitochondrial function and caused TM4 cells to undergo apoptosis and necrosis.

## INTRODUCTION

Besides providing energy and protein in animals’ nutrition, forages or other subsidiary agricultural products contain other nutrients and a wide variety of secondary metabolites. One group of secondary metabolites, tannins, are widely contained in sorghum (0.5 to 1.3 g/100 g, dry matter [DM]), grape (2 to 8 g/100 g in skin, and 13 to 15 g/100 g in seed, DM), and leguminous forages (0.4 to 11.6 g/100 g, DM) [1–3], and have long been the object of interest [4]. In livestock husbandry, tannins have been studied for their detrimental effects on herbivory, pathogens, parasites, or toxicity. Tannins also affect feed utilization and especially protect protein from fermentation including a shift in N excretion from urine to feces, and reduce ruminal methanogenesis [5,6].

Gallic acid (3,4,5-trihydroxybenzoic acid, GA), obtained by the hydrolysis of tannins, is a plant polyhydroxy phenol with well-known natural anti-oxidant ability [7]. As a strong natural anti-oxidant to scavenge reactive oxygen species (ROS), GA has been used as anti-oxidant food additive to inhibit oxidation of processed food products [8]. GA is easily absorbed in blood plasma after intake of tannins rich food [9]. Controversially, GA also is proposed to be pro-oxidant with its ability to inhibit superoxide dismutase and enhances ROS [8,9], and affects animal reproduction. To our knowledge, GA significantly decreases basal activities of 3β-hydroxysteroid dehydrogenases (HSD) and 17β-HSD in the testes and effects testosterone concentration leading to low sperm counts and quality in mice [10]. In mammals, Sertoli cells play a central role in sexual differentiation and in spermatogenesis. Taken together, we hypothesized that in males, GA may induce Sertoli cells apoptosis to affect spermatogenesis. Thus, the aim of this study was to investigate the effects of GA on apoptosis and necrosis of non-tumorigenic mice TM4 Sertoli cells via detecting the mitochondrial dysfunction, Cyclin B1, proliferating cell nuclear antigen (*PCNA*), Bcl-2-associated X protein (*BAX*), and Caspase-3 gene expression.

## MATERIALS AND METHODS

### Reagents

The Dulbecco’s modified Eagle’s medium (DMEM), fetal bovine serum (FBS), horse serum, trypsin-ethylenediaminetetraacetic acid solution and phosphate buffer saline (PBS) were purchased from Life Technologies (Carlsbad, CA, USA). BCA assay kit, goat anti-rabbit IgG antibody, rabbit anti-PCNA, rabbit anti-BAX, rabbit anti-Caspase-3, and rabbit anti-GAPDH were purchased from Bioss Biotechnology (Beijing, China). WST-1 kit was purchased from Roche (Mannheim, Germany). IP cell lysis buffer, JC-1 kit, beyoECL plus kit, apoptosis and necrosis assay kit were purchased from Beyotime Biotechnology (Nantong, China). Trizol reagent, primescript RT reagent kit with gDNA eraser and SYBR premix Ex Taq kit were purchased from Takara (Dalian, China). GA was purchased from Sigma (St. Louis, MO, USA). All other reagents were purchased from Sangon Biotech (Shanghai, China).

### Cell culture

The non-tumorigenic mice TM4 Sertoli cell line [11] was purchased from BioHermes (Wuxi, China) and was seeded as monolayer cultured in DMEM supplemented with 5% FBS and 2.5% horse serum at 37°C and 5% CO_2_ in humidified air. After completely attached, the cells were treated with pre-warmed fresh DMEM with or without GA for different experiments.

### Cell viability assays

To determine the effects of GA on TM4 cells viability and growth, TM4 cells (1×10^4^) were seeded in a 96-well plate per well and treated with GA (0, 20, 50, 100, 200, and 400 μM) according to previous studies [10,12] for 12 h and 24 h. At the end of the culture period, 10 μL WST-1 solution was added to each well, and incubated for 1.5 h. The absorbance was measured at 460 nm using ELX 808 microplate reader (BioTek, Winooski, VT, USA). The absorbance of culture medium plus WST-1 without cell was invoked as background control. TM4 cells viability were given in the relative percentage of the control group treated without gallic acid.

### Mitochondrial membrane potential assays

The mitochondrial membrane potential (MMP) detection was performed according to the manufacturer’s MMP JC-1 detection kit instructions. Non-damaged (healthy) mitochondria of high MMP were indicated by red fluorescence due to JC-1 staining and were accumulated in matrix of mitochondria to form a polymer as JC-1 aggregate. While in damaged cells, the MMP was breaking down and the JC-1 monomers were seen dispersed through the entire cell and indicated by the green fluorescence [13]. After 0, 20, and 400 μM GA treated for 24 h, TM4 cells were washed twice with PBS. One mL fresh culture medium/JC-1 working solution (1:1) was hereby added per well, and incubated for 20 min at 37°C. Then, TM4 cells were washed twice with JC-1 washing buffer. Subsequently, MMP was reviewed on a fluorescence microscope (Olympus, Tokyo, Japan) under blue and green light at the same exposure parameters, respectively. The red and green fluorescence images were merged using cellSens Standard software (Olympus, Japan).

### Hoechst 33342 and propidium iodide staining

After GA (0, 20, and 400 μM) treatment for 24 h in 24-well plates, cells were washed twice with cold PBS, then, incubated with 5 μL Hoechst 33342 and 5 μL propidium iodide (PI) in 1 mL staining solution for 20 min at 4°C. After that, cells were washed with PBS and nuclear morphological changes were observed using fluorescence microscope (Olympus, Japan) under ultraviolet light and green light at same exposure parameters, respectively. The dark blue cells with normal nuclei were counted as viable cells, while the bright blue and red cells as necrosis [14]. Total cells and necrotic cells were counted, and the percentages of survival cells were calculated.

### RNA isolation and real-time polymerase chain reaction

TM4 cells were collected after GA (0, 20, and 400 μM) treated for 24 h, and total RNA was isolated using Trizol reagent according to the literature. RNA quantity and quality were estimated by NanpDrop 2000 spectrophotometer (Thermo Scientific, Waltham, MA, USA). The first-strand cDNA was synthesized using primescript RT reagent kit with gDNA eraser from 2 μg total RNA after removing genome DNA. Proliferation and apoptosis related genes expression were detected with specific primers on the Agilent Mx3000P real-time system (Agilent, Santa Clara, CA, USA) using SYBR premix Ex Taq kit as follows: preheating at 94°C for 5 min; 35 cycles at 94°C for 5 s; and 60°C for 30 s. Fluorescence signals were collected at the endpoint of each extension step. At the end of each amplification, amplified products were subjected to a dissociation gradient to verify the amplification of single product denaturation at the anticipated temperature. Amplification was carried out as recommended by the manufacturer for a 20 μL reaction size contained 1 μL of cDNA, 5 pmol of each primer, 10 μL of SYBR enzyme mix. Primers (Table 1) were designed to span introns to avoid false results from genomic DNA amplification. Each cDNA product was tested in triplicate. The levels of *Cyclin B1*, *PCNA*, *BAX*, and *Caspae-3* were normalized against glyceraldehyde-3-phosphate dehydrogenase (*GAPDH*) levels as relative fold change (2^−ΔΔCt^).

### Western blotting analysis

Total protein was isolated from TM4 cells after treatment with GA (0, 20, 400 μM) for 24 h using IP cell lysis buffer. After centrifuged at 10,000×g at 4°C for 15 min, total protein concentrations were estimated by BCA assay. Approximately 20 μg proteins were separated by sodium dodecyl sulfate-polyacrylamide gel electrophoresis on a polyacrylamide gel and then transferred onto polyvinylidene fluoride membranes with a pore size of 0.45 μm. After blocking with 5% non-fat milk for 2 h at room temperature, membranes were incubated with primary antibodies (1:1,000 for GAPDH and 1:800 for others) overnight at 4°C. After that, membranes were washed 3 times with PBS containing 0.1% Tween 20, then, incubated for 1 h with HRP-conjugate goat anti-rabbit secondary antibody (1:5,000) at room temperature. After washed 3 times, the western blot images were captured by Tanon 5200 Chemiluminescent image systems (Tanon, Shanghai, China) using beyoECL plus kit.

### Statistical analysis

All experiments were repeated independently at least three times, and data were presented as mean±standard error of the mean. Statistical analysis was carried out with SPSS 13.0 program (SPSS, Chicago, IL, USA). Normality of distribution were analyzed by one-sample Kolmogorov-Smirnov test. The homogeneity of variance was analyzed by Lenven’s test. The significance was analyzed by one-way analysis of variance test and multiple comparisons between groups were compared with LSD analysis of variance. Less than 0.05 p value was considered statistically significant.

## RESULTS

### Effects of gallic acid on cell viability

**Figure 1:**
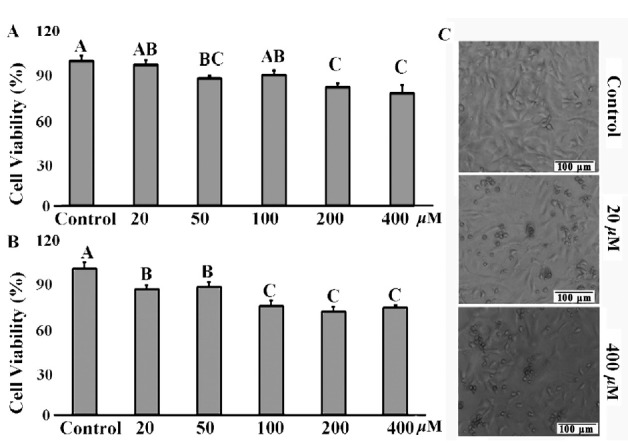
Cytotoxic effects of GA on mice TM4 cells. (A) and (B) Cell viability was determined by WST-1 assay after 20, 50, 100, 200, and 400 μM GA treated for 12 h and 24 h. (C) TM4 cell morphological changes were captured with Olympus microscope (100×) after 20 and 400 μM GA treated for 24 h. Photomicrographs were representatives of three independent experiments. GA, gallic acid. Data were expressed as mean (% of control group)±standard error of the mean of five independent experiments. Significant difference was defined as p<0.05, and the data with different capital letters in the same column show a significant difference.

Cell viability was significantly reduced by treatments with 200 and 400 μM GA for 12 h (Figure 1A). For 24 h treatments, GA significantly reduced cell viability compared with the control group (p<0.05) (Figure 1B) in a concentration dependent manner. The number of detached cells was increased in the GA treatment groups. Thus, the cellular density was decreased (Figure 1C). Based on these data, treatments with 20 and 400 μM for 24 h were selected for the subsequent experiments in this study.

### Mitochondrial dysfunction

**Figure 2:**
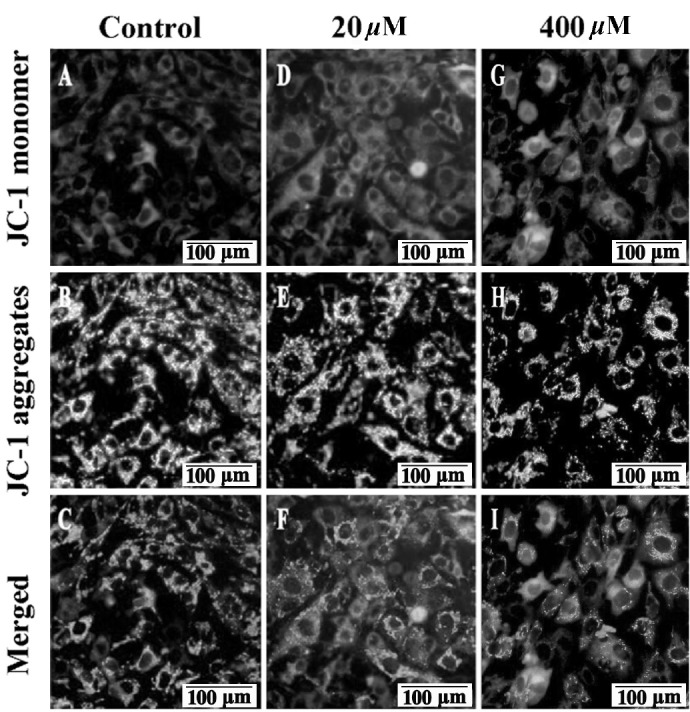
Effects of GA on mice TM4 cells mitochondrial membrane potential. Representative images of JC-1 stained TM4 cells were captured with Olympus microscope (100×) after 24 h treatment. High MMP in TM4 cells in control group emits strong red fluorescence (A) and pale green fluorescence (B). While, low MMP in 20 μM GA treated group (D and E) and in 400 μM (G and H) GA treated group was indicated by pale red fluorescence and strong green fluorescence, respectively. In the merged picture (C) of (A and B), (F) of (D and E) and (I) of (G and H), the cells shift from red-orange fluorescence to green fluorescence with a concentration dependent manner. GA, gallic acid; MMP, mitochondrial membrane potential. Bars indicate 100 μm.

MMP depolarization is generally perceived as an early sign of apoptosis. When MMP depolarization occurs, JC-1 staining shifts from JC-1 aggregates to JC-1 monomers (green fluorescence) [13]. As shown in Figure 2, compared to control group significant MMP depolarization was detected after 20 and 400 μM GA treatment, as demonstrated by the strong green fluorescence (Figure 2D, 2G) and pale red fluorescence (Figure 2E, 2H). Thus, these results clearly show that GA decreased TM4 cells’ MMP compared to control group with red-orange fluorescence (Figure 2C) to yellow-green (Figure 2F, 20 μM) and green (Figure 2I, 400 μM) in a concentration dependent manner.

### Cell necrosis and apoptosis assay

**Figure 3:**
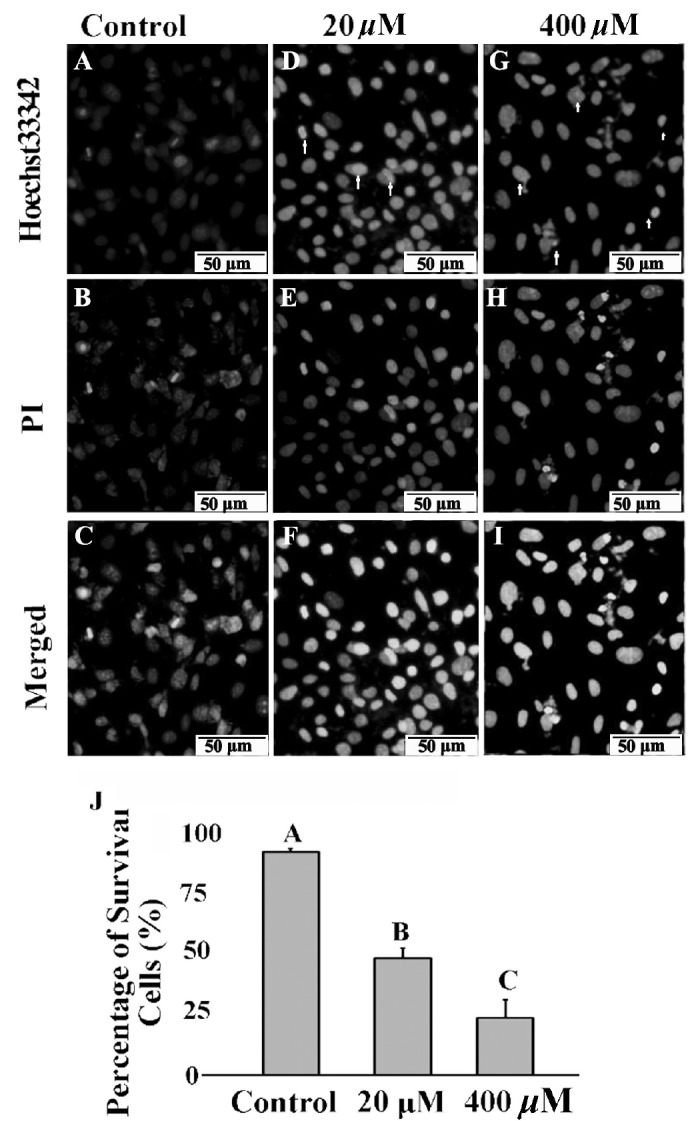
Effects of GA on mice TM4 cells necrosis and apoptosis. After PI/Hoechst 33342 staining, TM4 cells displayed pale blue and red fluorescence with Hoechst 33342 (A) and propidium iodide (PI) (B) stained in the control group. TM4 cells displayed strong blue and red fluorescence after 20 μM (D, E) and 400 μM (G, H) GA treated for 24 h. In the merged picture (C) of (A and B), (F) of (D and E) and (I) of (G and H), the cells appeared pink with a concentration dependent manner. Arrows indicate typical morphological feature of condensed and fragmented nuclei in apoptosis cells. Bars indicate 50 μm. Ratio of survival cells in different treatment were elevated (J). GA, gallic acid. Data were expressed as mean±standard error of the mean of three independent experiments. Significant difference was defined as p<0.05, and the data with different capital letters in the same column show a significant difference.

As shown in Figure 3, most of the normal control group cells were stained with few Hoechst 33342 and PI. After treatment with 20 and 400 μM GA, the number of nuclei stained with bright blue and red fluorescence showing late apoptosis or necrosis was significantly increased (Figure 3F, 3I). Apoptosis cells with condensed and fragmented nuclei after GA treatment were observed with Hoechst 33342 (Figure 3D, 3G; arrow). Based on the nuclear morphological changes after two dyes staining, the cells survival rates were significantly decreased from 93.1% (control) to 48.6% (20 μM, p<0.05), and 23.9% (400 μM, p<0.05) after GA treatment, respectively (Figure 3J).

### Effects of gallic acid on apoptosis-related genes expression

**Figure 4:**
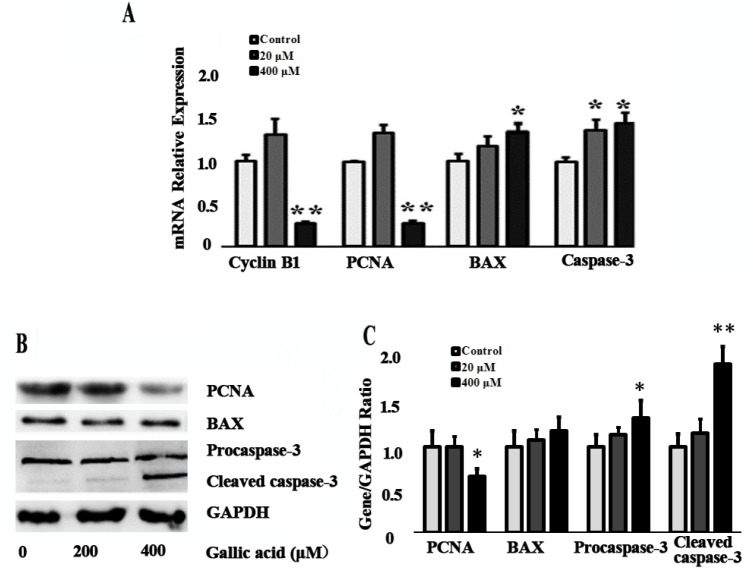
Effect of GA on the apoptosis related genes expression level. (A) The expression of Cyclin B1, PCNA, BAX, and Caspase-3 mRNA was determined by quantitative real-time polymerase chain reaction in mice TM4 cells after 20 and 400 μM GA treatment for 24 h. (B) The expression of PCNA, BAX, Proaspase-3 and Cleaved caspase-3 protein was determined by western blotting. GA, gallic acid; *PCNA*, proliferating cell nuclear antigen; *BAX*, Bcl-2-associated X protein; Data were expressed as mean±standard error of the mean of three independent experiments. Significant difference was identified as * p<0.05, ** p<0.01, compared with control group.

After 24 h treatments, 400 μM GA markedly inhibited Cyclin B1, PCNA mRNA expression and PCNA protein expression (p<0.05). However, as for apoptosis-related genes, *BAX* and *Caspase-3* mRNA were significantly up-regulated (p<0.05). Consistent with these results, Procaspase-3 and Cleaved caspase-3 protein also significantly up-regulated (p<0.05) after 400 μM GA treatment (Figure 4).

## DISCUSSION

Previous studies indicated that GA is easily absorbed in blood plasma and the concentrations of total metabolites reached 4 μM after ingestion of 50 mg GA rom Assam black tea [15]. *In vitro* studies, the 50% inhibitory concentration of GA for primary human pulmonary fibroblast cells is 300 μM [16]. Here, we evaluated the cytotoxic effects of 20 to 400 μM GA on mice TM4 cells. The results showed that the growth of TM4 cells was inhibited by GA in a concentration dependent manner, and the percentage of surviving cells declined after treatment with GA for 24 h. To our knowledge, other phenolic compounds such as Gossypol, Quercitrin occurring in fruits and vegetables have been found to induce pathological changes in testicular cells [17–19]. Interestingly, some phenolic compounds, such as Romarinic acid may have protective effects on the male reproductive system [20].

PCNA has been used as a biomarker in regulation of apoptosis. Cyclin B1 ensures the cells go through G2/M checkpoint via binding with cyclin-dependent kinase [21]. For example, down-regulation of *PCNA* and *Cyclin B1* gene expression in mouse spermatogonial GC-1 cells resulted in significantly increased apoptosis index and caused G2/M cell cycle arrest [22,23]. It has been shown that GA arrested cancer cells at the G2/M transition during cells cycle phase and induced apoptosis [24–26]. So, we did not determine the cell cycle in this study, based on 400 μM GA inhibited PCNA and Cyclin B1 expression.

In addition, the cytotoxic effect of GA on mice TM4 cells may associate with ROS accumulation. Our results showed that 400 μM GA significantly induced BAX expression. BAX is a pro-apoptotic member of the BCL2 family [23], its oligomerization and channel formation caused mitochondrial membrane permeabilization via inducing cytochrome c release [27,28]. Cytochrome c release results in mitochondrial uncoupling and ROS accumulation [29]. Previously studies demonstrated that gallic acid significantly increased intracellular H_2_O_2_ production, caused accumulation of ROS and resulted mitochondrial dysfunction, thus induced mice spermatogonia (GC-1), spermatocytes (GC-2), and TM3 Leydig cells apoptosis [12,30]. In this study, after GA treated, TM4 cells mitochondrial dysfunction was indicated by JC-1- monomers green fluorescence, which could promote cell apoptosis.

Caspase-3 expression were significantly changed and activated cleaved Caspase-3 was also detected. Cell shape and nuclear morphology were extremely altered during apoptosis. DNA damage was indicated using nuclei stained with bright blue and red fluorescence after incubation with Hoechast33342/IP staining [14], and the apoptosis and necrosis ratio increased as the dose of GA increased. Fragmented nuclei were also noted in TM4 cells in this study. These results indicated that GA triggered the caspase cascade and active caspase-3 caused DNA damage.

In Conclusion, the present work showed clearly that GA induced mice TM4 cells apoptosis and necrosis. Furthermore, GA treatment inhibited PCNA and Cyclin B1 expression and inhibited TM4 cells growth. Moreover, GA induced mitochondrial membrane permeabilization, triggered the caspase cascade, and cleaved Caspase-3 activation, then, caused DNA damage and TM4 cells apoptosis or necrosis.

## Figures and Tables

**Table 1:** List of primers used for quantitative real-time polymerase chain reaction

Gene (ID)	Primer sequence 5′-3′	Fragment length (bp)	Annealing temperature (°C)
*GAPDH*	F: AGGTCGGTGTGAACGGATTTG		
XM_017321385.1	R: TGTAGACCATGTAGTTGAGGTCA	123	60
*Caspase-3*	F: AGCAGCTTTGTGTGTGTGATTCTAA		
XM_017312543.1	R: AGTTTCGGCTTTCCAGTCAGAC	137	60
*BAX*	F: CAGGATGCGTCCACCAAGAA		
XM_011250780.2	R: GCAAAGTAGAAGAGGGCAACCA	197	60
*PCNA*	F: TAAAGAAGAGGAGGCGGTAA		
NM_011045.2	R: TAAGTGTCCCATGTCAGCAA	175	60
*Cyclin B1*	F: AGATGCAGTTGGCACCATGT		
NM_172301.3	R: TTCGACAACTTCCGTTAGCCT	148	60

*GAPDH*, glyceraldehyde-3-phosphate dehydrogenase; *BAX*, Bcl-2-associated X protein; *PCNA*, proliferating cell nuclear antigen.
